# Iron and Insomnia in Autism Spectrum Disorder

**DOI:** 10.15844/pedneurbriefs-34-17

**Published:** 2020-12-09

**Authors:** Innessa Donskoy, Darius Loghmanee

**Affiliations:** 1 Advocate Children’s Hospital, Park Ridge, IL

**Keywords:** Autism, Insomnia, Restless Legs Syndrome, Iron, Ferrous Sulfate

## Abstract

Investigators from four major Universities studied the impact of iron supplementation on insomnia symptoms in children with Autism Spectrum Disorder (ASD) and ferritin levels not indicative of iron deficiency anemia.

Investigators from four major Universities studied the impact of iron supplementation on insomnia symptoms in children with Autism Spectrum Disorder (ASD) and ferritin levels not indicative of iron deficiency anemia. The study assessed twenty children who had confirmed ASD, difficulties with sleep onset or maintenance, and ferritin levels 17-50 ng/mL. [1]

COMMENTARY. Sleep disorders are prevalent in children with ASD and have tremendous implications on their physical and social-emotional health [2]. In addition to strengthening positive behavioral patterns surrounding sleep, pediatric neurologists must also consider organic sleep disorders that contribute to sleep challenges. This study focused on children with insomnia and ferritin levels <50 ng/mL, a value at which treatment with iron repletion is indicated for Restless Legs Syndrome (RLS) or Periodic Limb Movement Disorder (PLMD) in children [3]. The aim was to assess if difficulties with sleep onset or maintenance would improve with empiric treatment of a ferritin <50 ng/mL without a formal sleep disorder. While empiric therapy was not shown to be effective in managing insomnia in the setting of ASD, it highlights the importance of evaluating for at least two independent sleep disorders that may also present with difficulties falling or staying asleep: RLS and PLMD [1].

A reported history of uncomfortable sensations, often in the legs, diagnoses RLS, which are associated with the urge to move and are 1) worse at night, 2) worse at rest, and 3) relieved by movement [3]. Ferritin levels are often empirically treated when <50 ng/mL. The lack of an improvement in sleep onset after empiric treatment in this study suggests that the sample may primarily represent children with ASD who do not have RLS. Hopefully, our ability to diagnose RLS in children with ASD will improve in the future to allow for more targeted treatment.

PLMD is diagnosed in children with polysomnography demonstrating >5 limb movements/hour with impairment in daytime functioning [4]; a “wild sleeper” to a parent. Parental observations may also represent Restless Sleep Disorder (RSD), who has subjective reports of sleep movements but no polysomnographic evidence of PLMD [5]. Early findings suggest that these children have decreased iron stores, as well [5]. Arousals with limb movements may also lead to prolonged awakenings if re-initiating sleep is a challenge [6]. Iron testing/treatment follows the RLS pathway. However, as used in this study, actigraphy is not validated to diagnose or assess the response to treatment in PLMD [7]. As with RLS, children with PLMD (or RSD), who could be responders to iron, must be appropriately identified before making therapeutic decisions.

Sleep-focused history taking and formal sleep testing, when indicated, will help identify patients with ASD who could benefit from iron testing and supplementation for the treatment of specific sleep disorders. Selecting these patients out of future studies will help clarify what role, if any, iron can play in managing sleep in this complex population.

## Disclosures

The authors have declared that no competing interests exist.
